# ZCCHC8 p.P410A disrupts nucleocytoplasmic localization, promoting idiopathic pulmonary fibrosis and chronic obstructive pulmonary disease

**DOI:** 10.1186/s10020-024-00913-9

**Published:** 2024-09-10

**Authors:** Chen-Yu Wang, Si-Hua Chang, Cheng-Feng Hu, Yi-Qiao Hu, Hong Luo, Lv Liu, Liang-Liang Fan

**Affiliations:** 1 Department of Pulmonary and Critical Care Medicine, Research Unit of Respiratory Disease, Hunan Diagnosis and Treatment Center of Respiratory Disease, the Second Xiangya Hospital, Central South University, Changsha, 410011 China; 2 Department of Cell Biology, School of Life Sciences, Central South University, Changsha, 410013 China

**Keywords:** ZCCHC8 mutation, Idiopathic pulmonary fibrosis, Chronic obstructive lung disease, Nuclear exosome-targeting complex

## Abstract

**Background:**

Idiopathic pulmonary fibrosis (IPF) is a special kind of chronic interstitial lung disease with insidious onset. Previous studies have revealed that mutations in ZCCHC8 may lead to IPF. The aim of this study is to explore the ZCCHC8 mutations in Chinese IPF patients.

**Methods:**

Here, we enrolled 124 patients with interstitial lung disease from 2017 to 2023 in our hospital. Whole exome sequencing and Sanger sequencing were employed to explore the genetic lesions of these patients.

**Results:**

Among these 124 patients, a novel mutation (NM_017612: c.1228 C > G/p.P410A) of *Zinc Finger CCHC-Type Containing 8* (*ZCCHC8*)was identified in a family with IPF and chronic obstructive lung disease. As a component of the nuclear exosome-targeting complex that regulates the turnover of human telomerase RNA, *ZCCHC8* mutations have been reported may lead to IPF in European population and American population. Functional study confirmed that the novel mutation can disrupt the nucleocytoplasmic localization of ZCCHC8, which further decreased the expression of DKC1 and RTEL1, and finally reduced the length of telomere and led to IPF and related disorders.

**Conclusions:**

We may first report the ZCCHC8 mutation in Asian population with IPF. Our study broadens the mutation, phenotype, and population spectrum of ZCCHC8 deficiency.

**Supplementary Information:**

The online version contains supplementary material available at 10.1186/s10020-024-00913-9.

## Background

As the most common type of chronic interstitial lung diseases with insidious onset, idiopathic pulmonary fibrosis (IPF) clinically manifests as the formation of honeycomb lung fibrosis at subpleural and basal reticulation on the chest radiograph and histopathological as usual interstitial pneumonia (Liu et al. 2022; Podolanczuk et al. 2023). Most IPF patients presented with a gradual onset of exertional dyspnea and/or a nonproductive cough and died from respiratory failure three to five years after diagnosis (Liu et al. 2022). As a rare disorder, the prevalence of IPF is on the rise with more than 50,000 new cases diagnosed annually (Maher et al. 2021). However, the optimal medical therapy for the treatment of idiopathic pulmonary fibrosis has yet to be identified. Unless there are contraindications, lung transplantation is the most effective treatment for IPF at present (Karampitsakos et al. 2023).

It is widely accepted that both heredity and environment elements play a crucial role in the occurrence and development of IPF (Moss et al. 2022). For example, familial pulmonary fibrosis accounts for 5–20% of IPF cases, with up to one-third of sporadic IPF cases having a family history of pulmonary fibrosis (Podolanczuk et al. 2023). Simultaneously, certain viral infections, air pollution, and some exposures in the workplace may also be risk factors for IPF (O’Dwyer et al. 2019). Previous studies have revealed that variants in several genes encoding telomere biology-related proteins and surfactant proteins are the major genetic factors for IPF (Allen et al. 2020; Revy et al. 2023). Recently, variants in more than 10 telomere biology-related genes have been proved can promote the risk of IPF overtly, such as *telomerase reverse transcriptase* (*TERT*), *telomerase RNA component* (*TERC*), *dyskerin* (*DKC1*), *regulator of telomere elongation helicase 1* (*RTEL1*) et al. (Allen et al. 2020; Revy et al. 2023). However, there were only one or two mutations of some telomere biology-related genes were reported in IPF patients, such as *Zinc Finger CCHC-Type Containing 8* (ZCCHC8) gene (Gable et al. 2019), which makes the difficulties to understand the relationship between these genes and IPF.

AS a scaffold protein, ZCCHC8 can interact with Mtr4 exosome RNA helicase (MTR4) and RNA binding motif protein 7 (RBM7) to form the nuclear exosome-targeting (NEXT) complex, which contributes to the exosomal degradation of non-coding promoter-upstream transcripts, enhancer RNAs and 3’-extended products of histone- and small nuclear RNA transcription (Puno and Lima 2022; Tseng et al. 2015). Hence, as a part of NEXT complex, ZCCHC8 is important for the maturation of telomerase RNA (Tseng et al. 2015; Falk et al. 2016; Puno and Lima 2018). In *ZCCHC8* knock out HeLa cell line, the levels of matured telomerase RNA were reduced, and the activity of telomerase activity was also decreased (Gable et al. 2019). In 2019, Gable et al. first reported that mutations in *ZCCHC8* can lead to IPF and/or bone marrow failure syndrome (Gable et al. 2019). Since then, *ZCCHC8* was thought to be the causative gene of telomere biology disorders. At present, only two mutations of *ZCCHC8* were reported in three families with IPF and/or bone marrow failure syndrome (Gable et al. 2019; Groen et al. 2024; Nitschke et al. 2024).

Here, we analyzed 124 patients with interstitial lung disease by whole exome sequencing and Sanger sequencing. A novel mutation (NM_017612: c.1228 C > G/p.P410A) of *ZCCHC8* was identified in a Chinese family with IPF and chronic obstructive lung disease (COPD). Telomere length detection and functional studies in vitro revealed that the mutation disrupted the expression and nucleocytoplasmic localization of ZCCHC8, further decreased the expression of DKC1 and RTEL1, and reduced the length of telomeres.

## Methods

### Subjects

Totally of 124 patients who were diagnosed with interstitial lung disease in the Second Xiangya Hospital participated in the study (Table S1) (Guo et al. 2023). Blood was obtained from each patient and related family members. High-resolution computed tomography (HRCT), pulmonary function and other clinical data including serological antibody detection for tuberculosis and lung cancer were also collected.

### Genetic analysis

The genomic DNA was extracted from peripheral blood lymphocytes of all the participates with a QIAamp DNA Blood Midi Kit (Qiagen51183, QIAGEN, Germany). The BerryGenomics Biotech Company (Beijing, China) is responsible for the whole-exome sequencing and regular filtering analysis of the proband (II-5). The strategies of data filtering referred to our previous study (Liu et al. 2023a, b, 2024). Polymerase chain reaction (PCR) with designed primers (primer sequences will be provided upon requests) was performed in a BioRad-T100 PCR machine (Bio-Rad, USA), and the products were sequenced by an ABI 3100 Genetic Analyzer (ABI, USA).

### Bioinformatic analysis

The conservation analysis was performed by comparing amino acid sequences of ZCCHC8 among different species. The structure of the ZCCHC8 protein was built by Alphafold (https://alphafold.com) (Jumper et al. 2021; Varadi et al. 2024) and analyzed by Swiss-Model online software (https://swissmodel.expasy.org). The nuclear localization signal (NLS) of ZCCHC8 was predicted by DeepLoc-1.0 (https://services.healthtech.dtu.dk/services/DeepLoc-1.0/).

### Functional studies

The wild-type ZCCHC8 CDS (NM_017612) with a C-terminal Flag-tag in the pEnter was designed. The p.P410A-ZCCHC8 missense mutation was constructed by the Mut Express II Fast Mutagenesis Kit (C214-01, Vazyme, China). A549 cells were transfected with 1 μg plasmids (WT and/or mutation) by Lipofectamine™ 2000 CD Transfection Reagent (12566014, Invitrogen™, UAS). The cells were maintained at 37 °C, 5% CO_2_–controlled atmosphere in Dulbecco’s modified Eagle’s medium including 10% fetal bovine serum, 50 IU/mL penicillin, 50 mmol/L streptomycin, and glutamine.

For Immunofluorescent, the cultured cells were fixed with 4% paraformaldehyde and treated with 0.5% Triton X-100. The cells were stained with relevant antibodies and examined under the Leica SP5 platform according to standard methods. The antibody against Flag (ab205606) was purchased from Abcam (UK). Alexa Fluor 488 (A-11008) and DAPI (62247) were purchased from Thermo Fisher Scientific (USA).

For western blot, the protein was extracted using RIPA lysis buffer or NE-PER Nuclear and Cytoplasmic Extraction Reagents kit (78833, Thermo Fisher Scientific, USA) from A549 cell line transfected with wild-type or ZCCHC8 p.P410A mutation plasmids, as well as from peripheral blood lymphocytes of healthy controls (II-2 and II-6) and mutation carriers (II-1 and II-5). The protein concentration was measured by a Pierce™ BCA kit (23225, Thermo Fisher Scientific, USA). Totally of 30 μg protein was loaded per well. Bis-Tris NuPAGE gels (4–12%) from Invitrogen™ (EC6026BOX, Invitrogen™, USA) were applied to separate the protein by electrophoresis. After the related antibodies incubated, the chemiluminescent signals were scanned using a chemiluminescent imaging system (Alpha Innotech, USA). The antibodies against Histone H3 (sc-56616) and GAPDH (sc-32233) were purchased from Santa Cruz Biotechnology (USA). The antibody against DKC1 (ab156877) was purchased from Abcam (UK). The RTEL1 (PA5-72995) was purchased from Invitrogen™ (USA).

For telomere length detection, totally of 50 ng DNA was used to perform real-time PCR by employed a Biowing Telomere Detection Kit (Shanghai Biowing Applied Biotechnology Co., Ltd, China, include 1500 random peripheral blood samples data from Shaihai) according to established protocols (Sun et al. 2021). The Fast 7500 Real-Time PCR Systems (Applied Biosystems, USA) and 2^(−ΔΔCt)^ methods were applied to analyze the length of telomere length.

### Statistical analysis

Data were subjected to statistical analysis using Graph Pad Prism 8. The mean ± SEM were calculated based on at least three independent experiments. Two-tailed Student’s t‐tests and ANOVA were used for two‐group comparisons. Differences were considered statistically significant at *P* < 0.05, with significance indicated in figures as *P < 0.05, **P < 0.01 and ***P < 0.001.

## Results and discussion

**Fig. 1:**
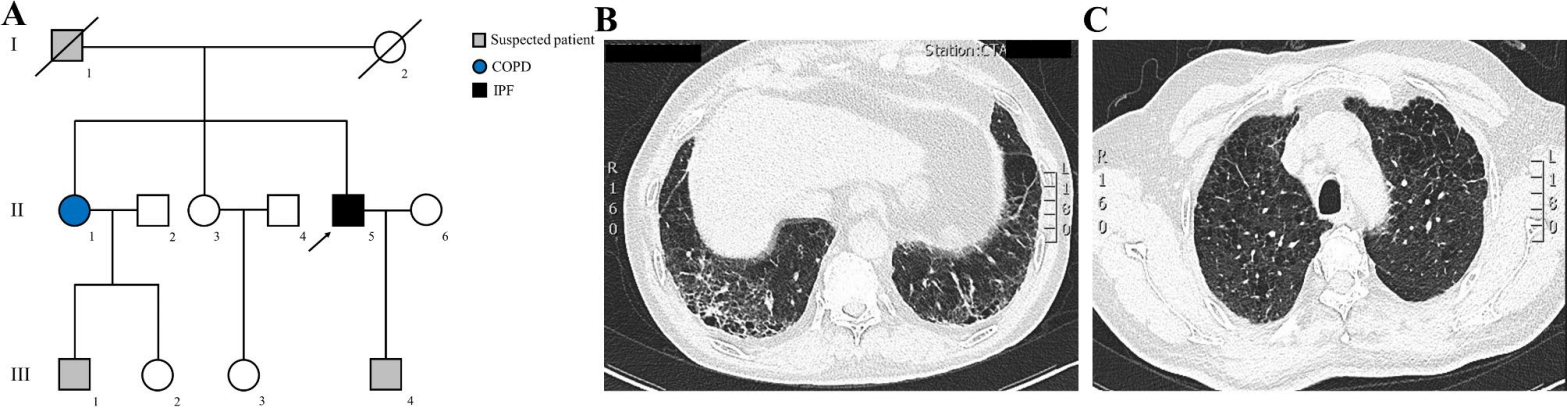
The family information and the clinic data. (**A**) Pedigree of the family affected with IPF and COPD. Family members are identified by generations and numbers. Squares indicate male family members; circles, female members; closed symbols, the affected members; open symbols, unaffected members; arrow, proband. The high-resolution CT of the proband (II-5) (**B**) and II-1 (**C**)

The proband (II-5), a 67-year-old male, suffered from repeated cough for 7 years, dyspnea for 1 year was admitted in our department (Fig. 1A). He had a smoking history for 50 years but denied occupational exposure. Pulmonary function test showed mild obstructive pulmonary ventilation impairment and severe impairment of diffusion function (Table 1). HRCT of the proband showed widespread reticular opacities, linear opacities, and subpleural predominantly honeycomb-like changes in the basal lung regions (Fig. 1B). The antibodies test for lung cancer, respiratory tract pathogens, comprehensive tuberculosis panel and connective tissue diseases were all negative. Finally, the patient was diagnosed as IPF based on the conclusions from multidisciplinary consultation (respiratory physician, radiologists and rheumatologists) on pulmonary interstitial diseases.

**Table 1:** The pulmonary function test of proband

	(measurement unit)	PRED	(PRE-RX)BEST	(PRE-RX) %PRED	(POST-RX) BEST	(POST-RX) %PRED
FVC	L	2.68	2.86	107	2.83	106
FEV1	L	2.13	1.50	71	1.55	73
FEV1/FVC	%	81	52		55	
TLC Sb	L	4.84	3.62	75		
RV Sb	L	1.97	0.84	43		
DLCO	Mmol/kPa.min	6.8	2.5	36		
DL Adj	Mmol/kPa.min	6.8	2.5	36		
DLCO/VA	DLCO/L	1.19	0.68	57		
DL/VA Adj	DLCO/L		0.68			

PRED = Prediction; PRE-RX BEST = best result previous therapy; POST-RX BEST = best result after therapy; FVC = Forced vital capacity; FEV1 = Forced expiratory volume in one second; TLC = Total lung capacity; RV = Residual volume; DLCO = Diffusion Capacity for Carbon Monoxide of the Lung; DL Adj = DLCO Adjustment; VA = Alveolar Ventilation

Family history investigation demonstrated that the proband’s father (I-1) had a history of hemoptysis and died from respiratory failure according to the description of the proband. The proband’s sister (II-1) was suffered from chronic obstructive lung disease (COPD) at 65 years old. HRCT showed increased transparency, disarranged broncho-vascular bundle structure, which consistent with emphysema of II-1 (Fig. 1C). In addition, his one nephew (III-1) was diagnosed as lung tuberculosis two years ago. And one son (III-4) of the proband claimed shortness of breath after general activities. Although his sone (III-4) refused to take further clinical testing due to far distance from our hospital, we still collected the peripheral blood via express delivery.

**Fig. 2:**
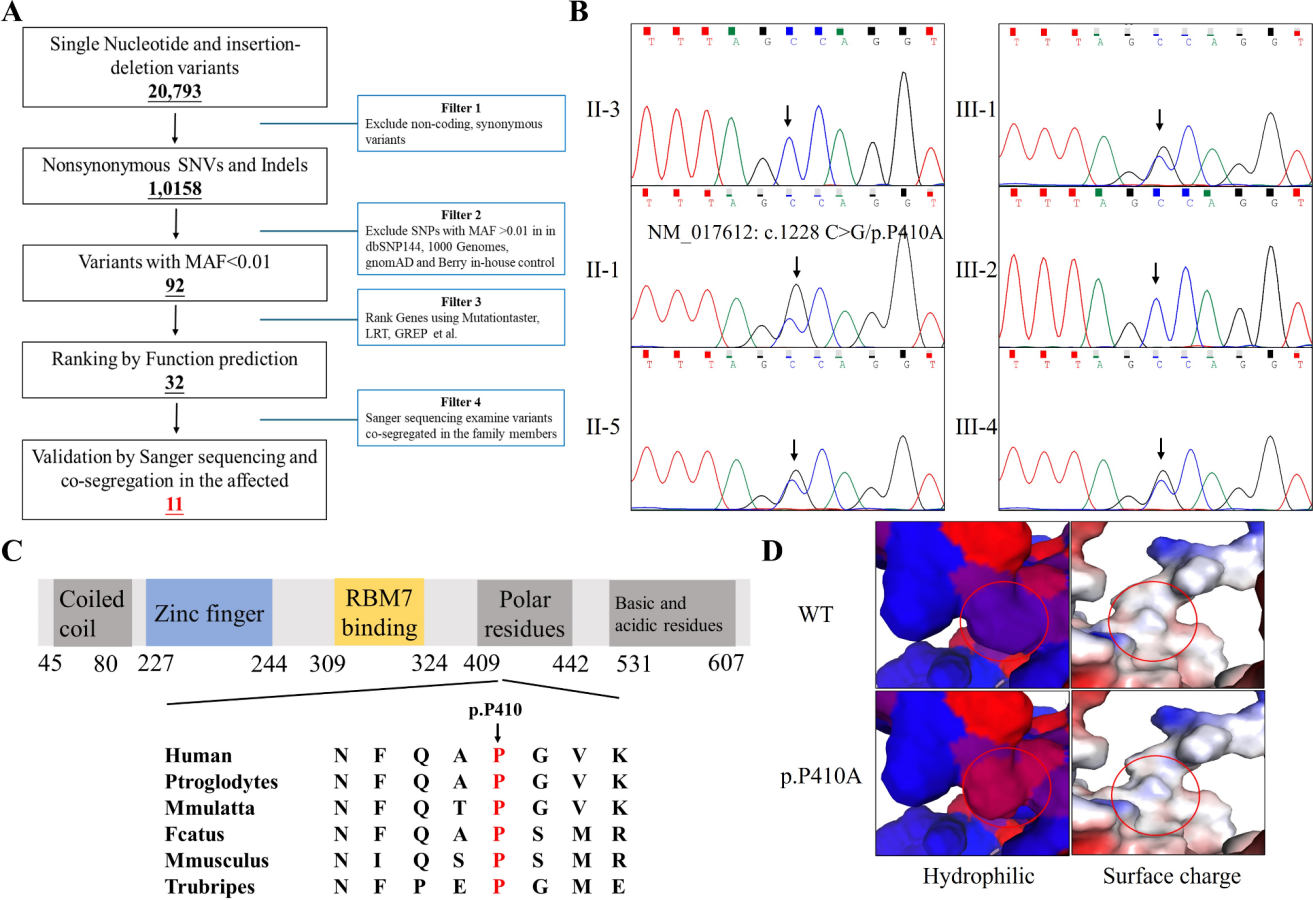
The genetic analysis of the family. (**A**) Schematic representation of the filter strategies employed in our study. (**B**) Sanger DNA sequencing chromatogram demonstrates the heterozygosity for a ZCCHC8 missense mutation (NM_017612: c.1228 C > G/p.P410A) in the family. (**C**) Alignment of multiple ZCCHC8 protein sequences across species. The P410 affected amino acid locates in the highly conserved amino acid region in different mammals (from Ensembl). Red column shows the P410 site. (**D**) The wild type ZCCHC8 (WT) protein structure and the mutant ZCCHC8 (p.P410A) protein structure were predicted by SWISS-MODEL online software. The hydrophilic surface area and surface charge and polarity of the WT and mutated ZCCHC8 were predicted

Whole exome sequencing was employed to analyze the genetic lesions of these 124 patients with interstitial lung disease. For the above-mentioned proband (II-3), whole-exome sequencing yielded 10.12 Gb of data with 99.5% coverage of target regions, and 97.4% of the target regions were covered over 10×. After alignment and single nucleotide variant calling, 20,793 variants were detected. After data filtering followed the methods in Fig. 2A, totally of 11 variants were remained and shown in Table 2. Among these 11 variants, the novel mutation (NM_017612: c.1228 C > G/p.P410A) of *ZCCHC8* was the most likely genetic lesion of the family presented IPF and COPD (Fig. 2B). The novel mutation, resulting in a substitution of proline by alanine, was located in a highly evolutionarily conserved site in polar residues domain of ZCCHC8 protein (Fig. 2C). Structural analysis further revealed that the p.P410A mutation changed the hydrophilic and surface charge of the ZCCHC8 protein (Fig. 2D).

**Table 2:** The mutation list of Sanger sequencing validation and co-segregation analysis

Chr	POS	RB	AB	Gene	Transcript
chr1	203667452	T	G	ATP2B4	NM_001684.4:p.Phe121Val/c.361T > G
chr3	52492768	A	G	NISCH	NM_007184.3:p.Lys90Glu/c.268 A > G
chr4	183522182	A	G	TENM3	NM_001080477.3:p.Asn206Ser/c.617 A > G
chr6	4044120	G	A	PRPF4B	NM_003913.4:p.Arg575Lys/c.1724G > A
chr9	79319733	T	C	PRUNE2	NM_015225.2:p.Glu2486Gly/c.7457T > C
chr11	89914856	G	A	NAALAD2	NM_005467.3:p.Val643Ile/c.1927G > A
chr12	122962505	G	C	ZCCHC8	NM_017612.4:p.Pro410Ala/c.1228G > C
chr14	74754915	C	A	ABCD4	NM_005050.3:p.Trp518Cys/c.1554 C > A
chr15	85165212	A	G	ZSCAN2	NM_181877.3:p.Ser596Gly/c.1786 A > G
chr17	29548917	A	G	NF1	NM_001042492.2:p.Asp564Gly/c.1691 A > G
chr18	44152076	G	A	LOXHD1	NM_144612.6:p.Pro674Ser/c.2020G > A

Chr: Chromosome; POS: position; RB: reference sequence base; AB: alternative base identified

**Fig. 3:**
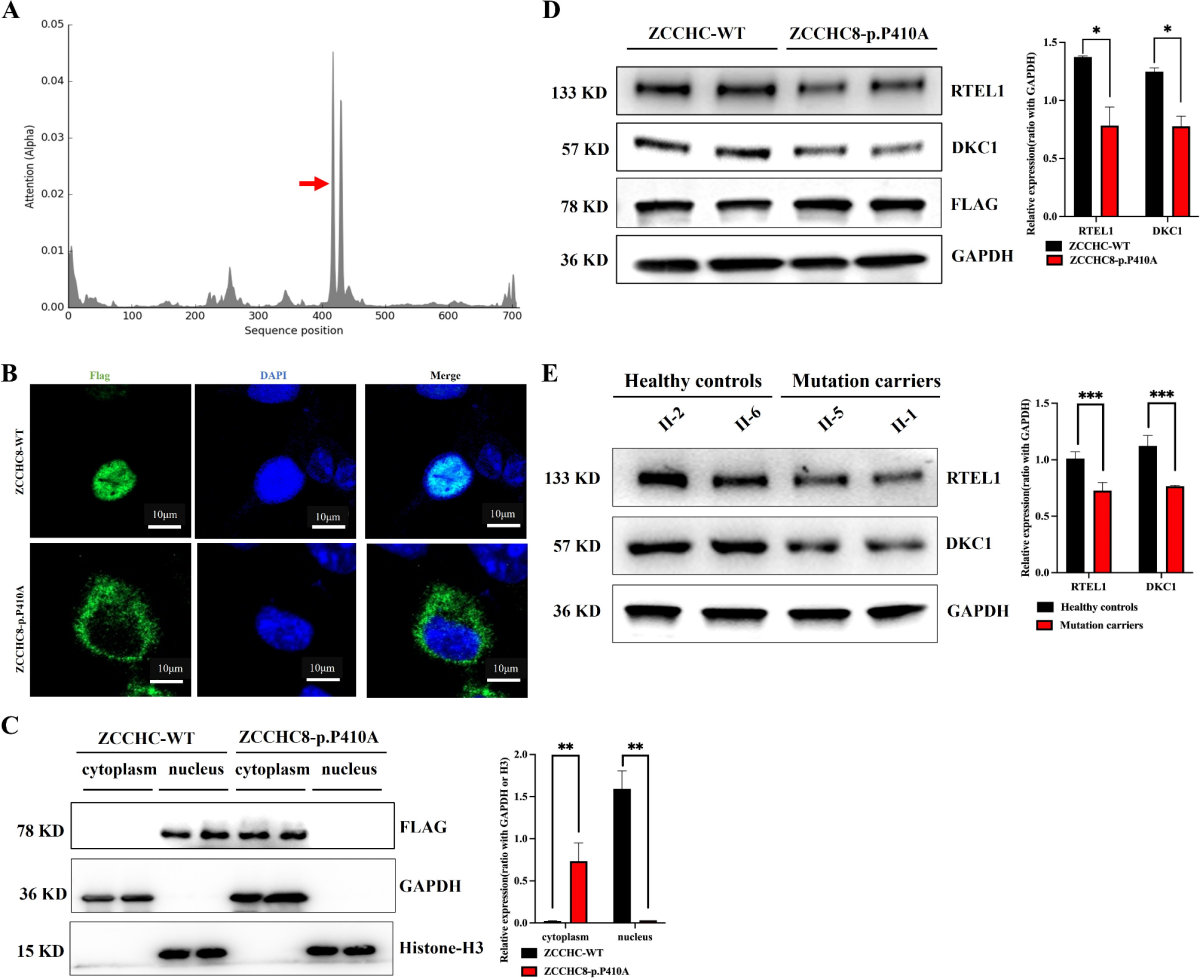
Functional study of the ZCCHC8 missense mutation. (**A**) DeepLoc-1.0 predicted that the p.P410A was in NLS domain of ZCCHC8. (**B**) Immunofluorescence staining showing the subcellular localization of WT and mutant ZCCHC8. Flag represents the transfected WT or mutant ZCCHC8 plasmids, and DAPI represents the cell nucleus. (**C**) Western blot analysis of the levels of transfected ZCCHC8 (Flag), GAPDH, and histone in the cytoplasm and nucleus from cells transfected with WT or mutant ZCCHC8 plasmids. (**D**) Western blot analysis of the levels of transfected ZCCHC8 (Flag), RTEL1, DKC1 and GAPDH from cells transfected with WT or mutant ZCCHC8 plasmids. (**E**) Western blot analysis of the levels of RTEL1, DKC1 and GAPDH in peripheral blood lymphocytes from healthy controls (II-2 and II-6) and mutation carriers (II-1 and II-5)

**Fig. 4:**
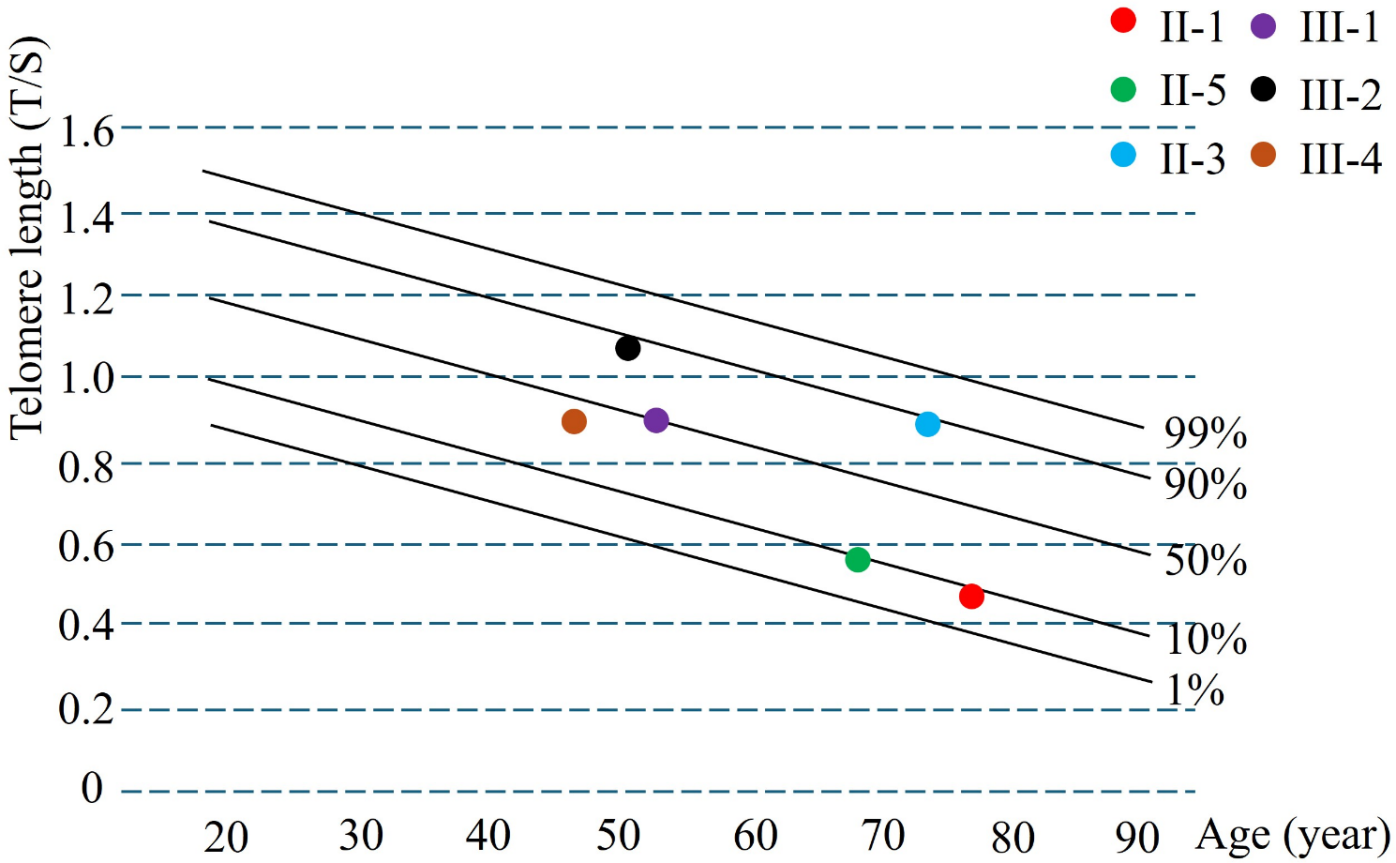
Telomere length of the mutation carriers (II-1, II-5, III-1 and III-4) and healthy family member (II-3 and III-2)

Because the DeepLoc-1.0 predicted that the novel mutation (p.P410A) was located in the nuclear localization signal (NLS) region of ZCCHC8 (Fig. 3A), we then constructed the WT and mutated (p.P410A) plasmids of ZCCHC8 and transfected them into A549 cell line (Figure S1). The immunofluorescence study showed that the WT ZCCHC8 was mainly in the cell nucleus but almost all the mutated (p.P410A) ZCCHC8 was in the cytoplasm (Fig. 3B), which was further confirmed by western blotting analysis of extracted nuclear and cytosolic proteins from the cells (Fig. 3C). Additionally, western blotting analysis also revealed that the mutation did not affect the expression of ZCCHC8 (Flag), but the expression of telomere biology-related genes such as *RTEL1* and *DKC1* was reduced obvious in the mutated (p.P410A) ZCCHC8 group compared to WT ZCCHC8 group (Fig. 3D), and the tendency were also confirmed in the peripheral blood mononuclear cells from p.P410A mutation carriers (II-1 and II-5) compared to healthy controls (II-2 and II-6) (Fig. 3E). Finally, the telomere length detection demonstrated that the length of telomeres in the healthy controls was remarkably longer than that in the mutation carriers (Fig. 4). Hence, functional studies confirmed that the novel mutation (p.P410A) located in the NLS region of ZCCHC8 which further disrupt the nucleocytoplasmic localization of ZCCHC8 and reduced the expression of telomere biology-related genes, and finally resulting in shorter telomere length and IPF.

Recently, an increasing number of studies revealed that genetic factors played a significant role in the occurrence and development of IPF (Revy et al. 2023). As a member of telomere biology-related genes, ZCCHC8 was involved in the senescence of type II alveolar epithelial cells (Gable et al. 2019). In 2019, Dustin L et al. first described the p.P186L mutation of *ZCCHC8* in patients with familial pulmonary fibrosis, and the same mutation was also identified in another study in patients with pulmonary fibrosis, hematological disease, and elevated liver enzymes (Gable et al. 2019; Nitschke et al. 2024). Recently, Karlijn et al. detected another new mutation (p. E196K) of *ZCCHC8* in patients with pulmonary fibrosis and other telomere-related phenotypes, such as pulmonary arterial venous malformations, emphysema, myelodysplastic syndrome, acute myeloid leukemia and dyskeratosis congenita, which confirmed that mutations of *ZCCHC8* may also lead to short-telomere syndromes (Groen et al. 2024). Here, we identified a novel mutation (NM_017612: c.1228 C > G/p.P410A) of *ZCCHC8* from 124 patients with interstitial lung disease. Family history survey revealed that the family members suffered from IPF and COPD. Functional studies showed that the novel mutation can disrupt the nucleocytoplasmic localization of ZCCHC8 and reduce the expression of known IPF pathogenic genes including *RTEL1* and *DKC1* which finally lead to shorter telomere length and IPF. Our discoveries were consistent with reported studies that loss of function of *ZCCHC8* in human and mice may result in short telomere syndromes manifested as familial IPF and progressive and fatal neurodevelopmental pathology with features of a ciliopathy (Gable et al. 2019; Groen et al. 2024; Nitschke et al. 2024). According to ACMG guideline (Richards et al. 2015), the p.P410A mutation of *ZCCHC8* is pathogenic: PS3 + PM1 + PM2 + PP1 + PP3.

Before the relationship between ZCCHC8 and IPF was established, ZCCHC8 was thought to be a glycogen synthase kinase that can act as fusion partner of ROS Proto-Oncogene 1 to drive the development of cancers including lung adenocarcinoma and glioblastoma (Gustafson et al. 2005). In 2011, Michal Lubas et al. first identified that the NEXT complex contained MTR4, RBM7 and ZCCHC8 (Lubas et al. 2011). Since then, thanks to the development of crystallization and structure determination, ZCCHC8 was found can interact with RBM7 via a proline-rich segment of ZCCHC8 (Falk et al. 2016; Puno and Lima 2018). The conserved C-terminal domain of ZCCHC8 can interact with MTR4 to stimulate MTR4 helicase and ATPase activities (Puno and Lima 2018). In 2022, M Rhyan Puno et al. determined cryogenic electron microscopy structures of NEXT complex and confirmed that ZCCHC8 can act as a scaffold to mediate the MTR4 helicase and the helicase core anchoring of RBM7 (Puno and Lima 2022). In addition, ZCCHC8 has also been found can interact with RNA binding motif protein encoded on the X chromosome (RBMX) to regulate the degradation of telomeric repeat-containing RNA (TERRA) (Liu et al. 2023a, b). At present, ZCCHC8 was recognized as a member of telomere biology-related genes which were responsible for telomere biology disorders including dyskeratosis congenita, IPF and Høyeraal–Hreidarsson syndrome et al. (Batista et al. 2022). Here, we identified a novel mutation (NM_017612: c.1228 C > G/p.P410A) of *ZCCHC8* in patients with IPF and COPD. This study reported the third pathogenic mutation of ZCCHC8 worldwide and was also first identified *ZCCHC8* mutation in Asian population. Our study expanded the mutation and population spectrum of ZCCHC8.

Previous studies have revealed that the deficiency of DKC1 and RTEL1 can lead to IPF (Revy et al. 2023). In this study, functional studies found that the novel mutation can reduce the expression of *RTEL1* and *DKC1*. The NEXT complex has been found can regulate the turnover of human telomerase RNA (Tseng et al. 2015). Hence, the p.P410A mutation that disrupted the nucleocytoplasmic localization of ZCCHC8 may result in deficiency of NEXT complex, which further affect the turnover of the human telomerase RNA. As a component of the telomerase complex (Sekne et al. 2022), the expression of DKC1 was reduced. As a T-loop assembly protein, RTEL1 was regulated by telomerase complex via interacting with telomere (Sarek et al. 2019). Therefore, level of RTEL1 was also decreased in ZCCHC8 mutated (p.P410A) group. The reduced expression of DKC1 and RTEL1 in ZCCHC8 mutated (p.P410A) group further confirmed that the p.P410A mutation of ZCCHC8 was the genetic lesion of the family with IPF and COPD. Simultaneously, the shorter telomere length in p.P410A mutation carriers also proved that the p.P410A mutation of ZCCHC8 may affect the telomerase complex.

Patients carried mutations of telomere biology-related genes often presented prominent genetic heterogeneity, especially the types of disease and age of onset (Revy et al. 2023; Kam et al. 2021). For example, previous studies revealed that mutations in RTEL1 can lead to isolated interstitial lung disease, Hoyeraal–Hreidarsson syndrome, bone marrow failure, ulcerative colitis and immunodeficiency, and the age of onset ranged from infancy to old age (Vannier et al. 2014; Hourvitz et al. 2024). Mutations of *ZCCHC8* were also identified in pulmonary fibrosis and short-telomere syndromes, which also showed typically genetic heterogeneity. In our study, the age of onset and the phenotypes among p.P410A mutation carriers were also different. We believed that environmental factors also contributed to the development of disorder. For example, the proband (III-5) presented symptoms at 59 years old, but his son (III-4), a 44-year-old young adult, has presented shortness of breath after general activities. Further investigation suggested that the proband had no history of cigarette smoking, but his son had a history of smoking for ten years. We don’t rule out the possibility that environmental factors such as smoking accelerated the progression of the disease for his son (Serrano Gotarredona et al. 2022). The COPD in the proband’s sister (II-1) may also affected by other environmental factors. Our study also expanded the phenotype spectrum of ZCCHC8 deficiency.

## Conclusions

In summary, by analyzing the whole exome sequencing data of 124 patients with interstitial lung disease, we identified a novel mutation (NM_017612: c.1228 C > G/p.P410A) of *ZCCHC8* in a Chinese family with IPF and COPD. Functional studies suggested that the novel mutation affected the transport of ZCCHC8 from the cytoplasm to the nucleus, which further decreased the expression of telomere biology-related genes including DKC1 and RTEL1, finally resulting in shorter telomere lengths and IPF. Our study may expand the mutation, phenotype, and population spectrum of ZCCHC8 deficiency and provide new insight into the role of the telomerase complex in IPF and related diseases.

## Electronic supplementary material

Below is the link to the electronic supplementary material.

Supplementary Material 1

## Data Availability

All the patients’ clinical information, raw WES mutation calling results and analyzing scripts for this study were deposited to a public GitHub repository (https://github.com/alfredsguo/ipf_wes_analysis). The clinical information and raw WES data have been encrypted for patient confidentiality. Access to the encrypted data is available from the corresponding author upon reasonable request.

## Abbreviations

COPD: Chronic obstructive lung disease
DKC1: Dyskerin
IPF: Idiopathic pulmonary fibrosis
MTR4: Mtr4 exosome RNA helicase
NEXT: Nuclear exosome-targeting
NLS: Nuclear localization signal
RNM7: RNA binding motif protein 7
RTEL1: Regulator of telomere elongation helicase 1
TERC: Telomerase RNA component
TERRA: Telomeric repeat-containing RNA
TERT: Telomerase reverse transcriptase
ZCCHC8: Zinc Finger CCHC-Type Containing 8
