# Supplementary material for: ZCCHC8 p.P410A disrupts nucleocytoplasmic localization, promoting idiopathic pulmonary fibrosis and chronic obstructive pulmonary disease
Source: Mol Med. 2024 Sep 10;30:144. doi: 10.1186/s10020-024-00913-9 (PMC11389302; doi:10.1186/s10020-024-00913-9)
Supplement: Supplementary file 1 — Supplementary Material 1 [file 10020_2024_913_MOESM1_ESM.docx]

ZCCHC8 p.P410A disrupts nucleocytoplasmic localization, promoting idiopathic pulmonary fibrosis and chronic obstructive pulmonary disease

Chen-Yu Wang^1,2#^, Si-Hua Chang^2#^, Cheng-Feng Hu^2#^, Yi-Qiao Hu^2^, Hong Luo^1^, Lv Liu ^1*^, Liang-Liang Fan ^1,2*^

^1.^ Department of Pulmonary and Critical Care Medicine, Research Unit of Respiratory Disease, Hunan Diagnosis and Treatment Center of Respiratory Disease, the Second Xiangya Hospital, Central South University, Changsha, 410011, China.

^2.^ Department of Cell biology, School of Life Sciences, Central South University, Changsha, 410013, China.

#contributed equally.

*Correspondence:

Lv Liu M.D.

e-mail: [docliulv@csu.edu.cn](mailto:docliulv@csu.edu.cn)

Department of Pulmonary and Critical Care Medicine, Research Unit of Respiratory Disease, Hunan Diagnosis and Treatment Center of Respiratory Disease, the Second Xiangya Hospital, Central South University, Changsha, China.

and

Liang-Liang Fan PH. D

e-mail: [swfanliangliang@csu.edu.cn](mailto:swfanliangliang@csu.edu.cn)

Department of Cell biology, School of Life Sciences, Central South University, Changsha, China.

**Supplementary material**

Table S1. The clinical characteristics of patients with interstitial lung disease.

| Characteristics | ILD cases (n=124) |
| --- | --- |
| Age | 62.88±11.06 |
| Gender |  |
| Male | 90 (72.58%) |
| Female | 34 (27.42%) |
| Smoking status |  |
| Former/current | 76 (61.29%) |
| Never | 48 (38.71%) |
| Clinical manifestation |  |
| Cough | 112 (90.32) |
| Dyspnea | 95 (76.61%) |
| Velco rales | 88 (70.97%) |
| Gastroesophageal reflux disease | 10 (8.06%) |
| Liver disease | 19 (15.32%) |
| Diabetes | 18 (14.51%) |

**
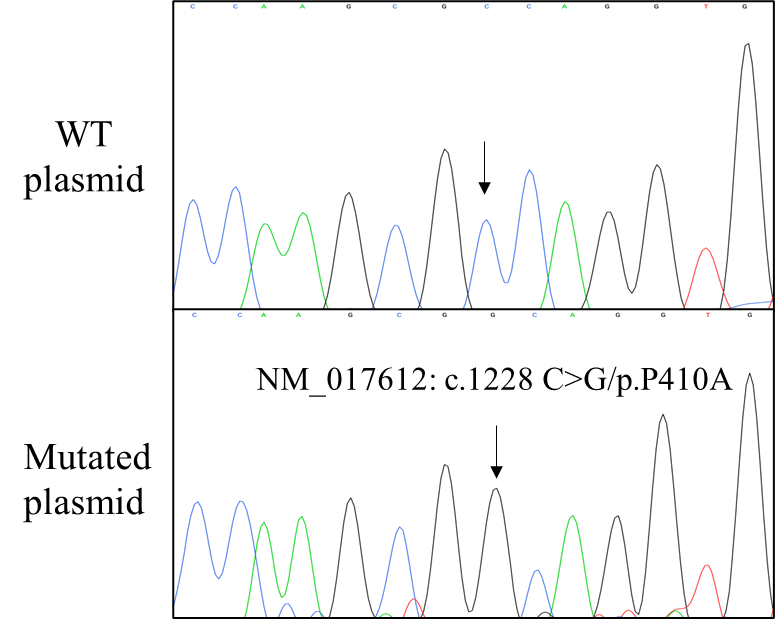
**

Figure S1. Sanger DNA sequencing demonstrates the ZCCHC8 missense mutation (NM_017612: c.1228 C>G/p.P410A) in the mutated plasmid.
